# Rethinking Implant Length: A Density‐Dependent Analysis of Primary Stability—An In Vitro Evaluation Using Resonance Frequency Analysis and Insertion Torque

**DOI:** 10.1155/ijod/4201028

**Published:** 2026-06-19

**Authors:** Sergio Alexandre Gehrke, Gracey Mosley, Juliana Campos Hasse Fernandes, Gustavo Vicentis Oliveira Fernandes

**Affiliations:** ^1^ Department of Pharmaceutical Science, School of Health Sciences, Vale do Itajai University (UNIVALI), Itajai, 88302–901, Brazil; ^2^ Department of Implantology, Bioface/Catolica de Murcia University, Montevideo, 11100, Uruguay; ^3^ Missouri School of Dentistry and Oral Health, A. T. Still University, St. Louis, 63104, Missouri, USA, atsu.edu; ^4^ GF10 Foundation, St. Louis, 63014, Missouri, USA; ^5^ Center for Interdisciplinary Research in Health (CIIS) - Universidade Católica Portuguesa (UCP), Viseu, Portugal

**Keywords:** bone density, dental implants, implant length, implant stability quotient (ISQ), insertion torque, primary stability, resonance frequency analysis

## Abstract

**Purpose:**

Accurate assessment of the primary stability of dental implants is essential for predicting osseointegration and determining loading protocols. This in vitro study aimed to evaluate the primary stability of dental implants of varying lengths across simulated bone densities using the insertion torque value (ITV) and resonance frequency analysis (RFA).

**Materials and Methods:**

Morse‐taper implants (5.0 mm diameter) were divided into four groups based on length: 5, 6, 7, and 8 mm. Implants were placed into polyurethane foam blocks of varying densities: PCF 40, PCF 30 (with 2 and 1 mm cortical layers), and PCF 20 (with a 1 mm cortical layer). Primary stability was measured via ITV and RFA, yielding an implant stability quotient (ISQ).

**Results:**

In high‐density foam (PCF 40), both ITV and ISQ increased proportionally with implant length, with the 8 mm implant demonstrating the highest stability (ITV = 109.26 ± 4.79 Ncm and ISQ = 73.5 ± 2.32). Conversely, in low‐density foam (PCF 20/1 mm), a direct relationship was observed, where the shortest implant (5 mm) exhibited the lowest stability (ITV = 36.8 ± 3.27 Ncm and ISQ = 45.8 ± 2.66), whereas the highest stability was presented by the 8 mm implant (ITV = 61.36 ± 2.00 Ncm and ISQ = 56.3 ± 2.67).

**Conclusion:**

Implant length significantly influences primary stability, but its effect is highly dependent on bone density. While shorter implants provide sufficient primary stability and may reduce surgical risks in highly dense bone, increased implant length is crucial for achieving superior primary stability in poor‐quality bone with thin cortical plates.

## 1. Introduction

Dental implants have revolutionized modern dentistry by providing a reliable solution for replacing missing teeth. The success of an implant depends on its ability to achieve and maintain stability within the maxillary or mandibular bone [1]. Implant stability is critical for osseointegration and is divided into two phases: primary and secondary stability [2]. Primary stability is achieved immediately after implant placement through mechanical retention, while secondary stability is reached over time as new bone forms around the implant. Therefore, accurately assessing implant stability is essential for ensuring long‐term success, reducing overall treatment time, and guiding clinical decisions, such as immediate or early loading protocols [3, 4].

Resonance frequency analysis (RFA) is one of the most widely accepted and noninvasive techniques for this assessment. This technique quantifies implant stability using the implant stability quotient (ISQ), with higher values indicating greater stability [5]. The reliability of the Osstell device has been demonstrated across various clinical settings, making it a standard tool for assessing stability without requiring direct contact with the implant‐bone interface. In addition to RFA, insertion torque value (ITV) is employed to measure the resistance encountered during placement, providing immediate insights into mechanical stability [6].

Recent studies have highlighted the paramount importance of bone density and quality in determining this primary stability. Bone density, often assessed via cone beam computed tomography (CBCT), significantly influences ISQ values [7]. Furthermore, factors, such as bone quantity and cortical thickness, play a crucial role in implant stabilization [8, 9]. The impact of implant macrogeometry on stability has also been a focal point of recent research; specific thread designs and lengths can enhance stability in varying bone densities [10, 11]. However, the exact interplay between the implant length and varying cortical thicknesses remains under investigation [12].

Understanding how the device performs in different scenarios is essential, especially in cases involving variable bone quality and density. This in vitro study aims to evaluate the primary stability of dental implants of varying lengths placed in simulated bone of different densities and cortical thicknesses, utilizing both ITV and RFA measurements. The null hypothesis tested was that there would be no significant difference in primary stability, as measured by ITV and RFA, among implants of varying lengths across different simulated bone densities.

## 2. Materials and Methods

This study followed the CRIS guidelines for in vitro studies.

### 2.1. Implant Characteristics

The study used Implacil de Bortolli dental implants (São Paulo, Brazil), all with a uniform diameter of 5.0 mm. The implants featured a standardized tapered macrogeometry, V‐shaped threads, and a sandblasted and acid‐etched surface topography with titanium oxide microparticles (~120 µm). The implants were divided into four experimental groups based on their length: (1) Group A: 5.0 mm ø × 5 mm length; (2) Group B: 5.0 mm ø × 6 mm length; (3) Group C: 5.0 mm ø × 7 mm length; and (4) Group D: 5.0 mm ø × 8 mm length (Figure S1).

### 2.2. Simulated Bone Substrate

To standardize the testing environment and isolate the variables of bone density and cortical thickness, synthetic polyurethane foam blocks (Sawbones, Nacional, São Paulo, Brazil) were utilized. Density was categorized in pounds per cubic foot (PCF). The blocks simulate the mechanical properties of human bone and were categorized into four distinct densities: (1) PCF 40: high density, representing type D1 solid rigid bone; (2) PCF 30/2 mm: medium density with a 2 mm simulated cortical layer, representing type D2 bone; (3) PCF 30/1 mm: medium density with a 1 mm simulated cortical layer; and (4) PCF 20/1 mm: low density with a 1 mm simulated cortical layer, representing poor‐quality type D3/D4 bone (Figure S2).

### 2.3. Measurement Protocols

**Figure 1:**
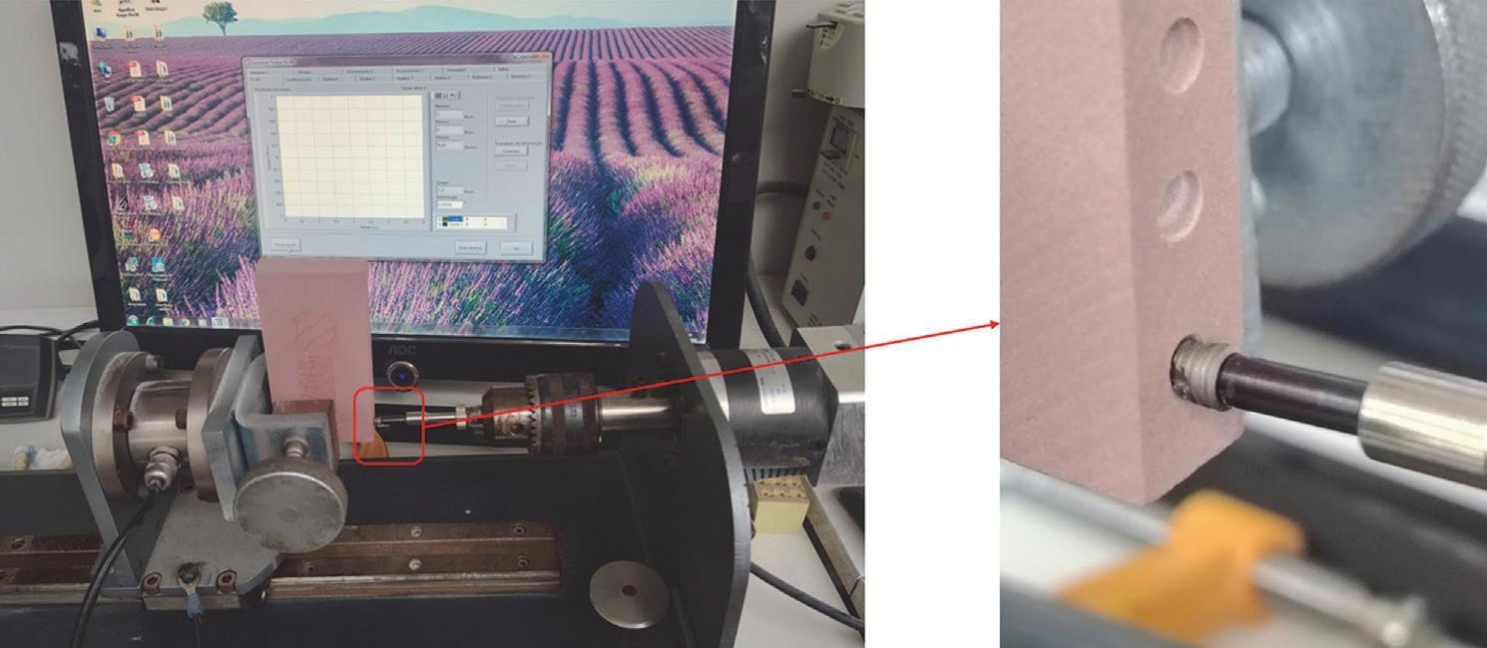
Automated insertion torque measurement setup. The experimental setup depicts a specialized motor system connected to a computer. An implant is being inserted into a bone block while the software records real‐time torque values (Ncm) in a graphical format.

The osteotomies were prepared using a surgical motor set to a drilling speed of 1000 RPM (BLM 600 Surgical implant device and 20:1 contra‐angle – Driller, Sao Paulo, Brazil), with copious irrigation of saline solution. Implants were placed into the polyurethane blocks according to the manufacturer’s drilling protocol (Figure 1). Ten samples (*n* = 10) were placed for each implant length within each bone density category, based on a power analysis derived from a previous similar in vitro study, designed to achieve 80% power at an alpha level of 0.05 [13].

**Figure 2:**
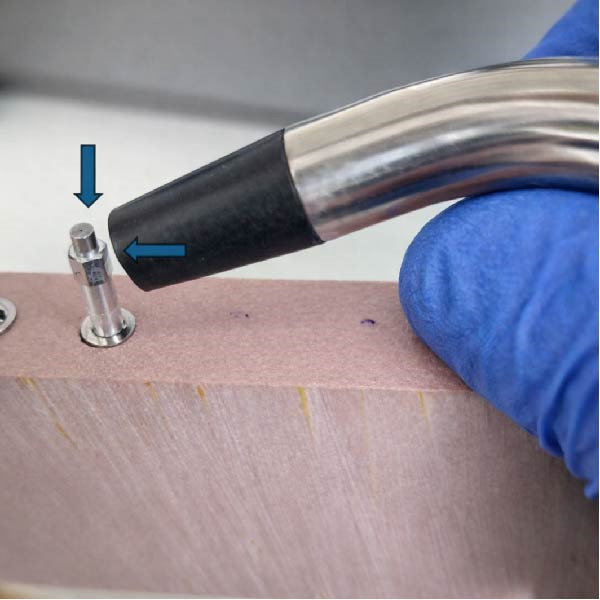
Resonance frequency analysis (RFA) for stability. The secondary stability metric was measured using an Osstell device. A magnetic SmartPeg is attached to the implant, and the probe measures the implant stability quotient (ISQ) through noncontact resonance frequency analysis.

The ITV, which measured the maximum torque required to fully seat the implant, was recorded in Ncm during the final phase of placement, and the RFA was measured immediately following implant placement. Primary stability was assessed utilizing an Osstell Mentor Device (Integration Diagnostic AB, Savadelen, Sweden). SmartPegs number 49 (Integration Diagnostic AB, Savadelen, Sweden), compatible with the implant system, were attached, and measurements were recorded in orthogonal directions to yield a final mean ISQ value for each implant (Figure 2). The ISQ ranged from 0 to 100 (measured between 3500 and 85,000 Hz).

The insertion torque was continuously recorded using a calibrated automated testing machine (CME‐30 nm, Técnica Industrial Oswaldo Filizola, Sao Paulo, Brazil) controlled by customized software (DynaView Torque Pro – v. 1.1.0). To ensure exact reproducibility, all insertions were performed under identical automated conditions at a standardized continuous insertion speed of 30 RPM until the implant was fully seated.

### 2.4. Statistical Analysis

Data were categorized by bone group (PCF 40, 30/2, 30/1, and 20/1 mm) and insertion depth (5–8 mm). Peak torque and ISQ were analyzed using descriptive statistics and a two‐way ANOVA to evaluate the main effects and their interactions. Tukey’s HSD test was used for post‐hoc pairwise comparisons.

The relationship between insertion torque and stability was assessed using Pearson’s correlation coefficient (*r*). Correlations were calculated both globally and within individual experimental groups to determine if the relationship remained consistent across conditions. All statistical analyses were performed using SPSS version 29.0 (IBM Corp., Armonk, NY, USA)/GraphPad Prism version 10 (GraphPad Software, Boston, MA, USA). The level of significance was set at *α* = 0.05.

## 3. Results

The primary stability measurements, categorized by bone density and implant length, are summarized in Table 1.

**Table 1:** Mean insertion torque values (ITV) and implant stability quotients (ISQ) ± standard deviation across varying bone densities and implant lengths.

Category/bone substrate	Measurement/source	5 mm	6 mm	7 mm	8 mm
PCF 40	ITV (Ncm)	71.72 ± 3.38	90.86 ± 4.18	102.24 ± 4.52	109.26 ± 4.79
	ISQ	72.00 ± 2.11	73.20 ± 2.25	73.40 ± 2.17	73.50 ± 2.32
PCF 30/2 mm	ITV (Ncm)	73.40 ± 2.59	74.10 ± 2.20	73.74 ± 3.24	75.36 ± 3.26
	ISQ	62.20 ± 2.30	62.20 ± 2.44	62.40 ± 3.20	62.60 ± 2.63
PCF 30/1 mm	ITV (Ncm)	70.70 ± 2.22	70.62 ± 2.44	71.26 ± 2.47	73.46 ± 2.01
	ISQ	60.30 ± 2.00	61.20 ± 2.82	61.90 ± 2.23	62.60 ± 2.80
PCF 20/1 mm	ITV (Ncm)	36.80 ± 3.27	51.58 ± 3.19	58.28 ± 2.90	61.36 ± 2.00
	ISQ	45.80 ± 2.66	52.00 ± 3.06	53.70 ± 2.54	56.30 ± 2.67

Statistical correlation	Source of variation	Torque (F)	Torque (*p*)	ISQ (*F*)	ISQ (*p*)
	Group (bone type)	1159.41	<0.001	462.21	<0.001
	Depth	209.98	<0.001	15.23	<0.001
	Interaction	60.86	<0.001	6.03	<0.001

### 3.1. High‐Density Bone (PCF 40)

In the densest substrate (PCF 40), a direct proportional relationship was observed between the implant length and primary stability. The ITV increased significantly from 71.72 ± 3.38 Ncm for the 5 mm implant to 109.26 ± 4.79 Ncm for the 8 mm implant. Similarly, ISQ values increased steadily with length, rising from 72.0 ± 2.11 (5 mm) to 73.5 ± 2.32 (8 mm).

### 3.2. Medium‐Density Bone (PCF 30)

Interestingly, in the medium‐density substrates, the implant length had a negligible effect on primary stability. In the PCF 30 substrate with a 2 mm cortical layer, ITV and ISQ values remained remarkably consistent across all implant lengths. ITV values ranged tightly between 73.4 ± 2.59 Ncm and 75.36 ± 3.26 Ncm, while ISQ values hovered between 62.2 and 62.6. A similar plateau effect was observed in the PCF 30 blocks with a thinner 1 mm cortical layer, where ITV ranged from 70.62 to 73.46 Ncm and ISQ ranged from 60.3 to 62.6.

### 3.3. Low‐Density Bone (PCF 20/1 mm)

In the lowest‐density substrate (simulating poor‐quality bone with a thin 1 mm cortical layer), the implant length proved critical for achieving stability. A strong positive correlation was observed: the shortest implant (5 mm) exhibited markedly lower, potentially inadequate stability, with an ITV of 36.8 ± 3.27 Ncm and an ISQ of 45.8 ± 2.66. However, as the implant length increased to 8 mm, stability progressively and significantly improved, reaching a much more clinically acceptable ITV of 61.36 ± 2.00 Ncm and an ISQ of 56.3 ± 2.67.

### 3.4. Torque and Stability (ISQ)

The variables, torque and ISQ, varied significantly across the experimental conditions (Table 1). PCF 40 consistently exhibited the highest values for both metrics, particularly at greater depths. Conversely, the PCF 20/1 mm group showed the lowest overall torque and stability values. The statistics revealed that the interaction between insertion depth and bone type was highly significant (*p*  < 0.001) for both torque and ISQ.

**Figure 3:**
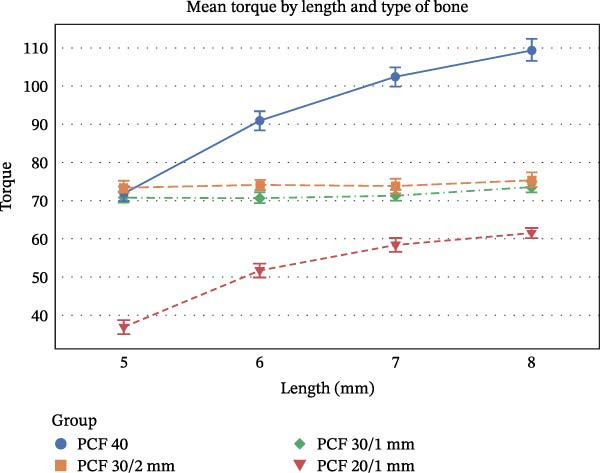
Mean torque values by bone density and implant length. This line graph illustrates that insertion torque increases proportionally with implant length. The 40 PCF bone (blue line) consistently required the highest torque, while the 20 PCF (yellow line) remained significantly lower.

**Figure 4:**
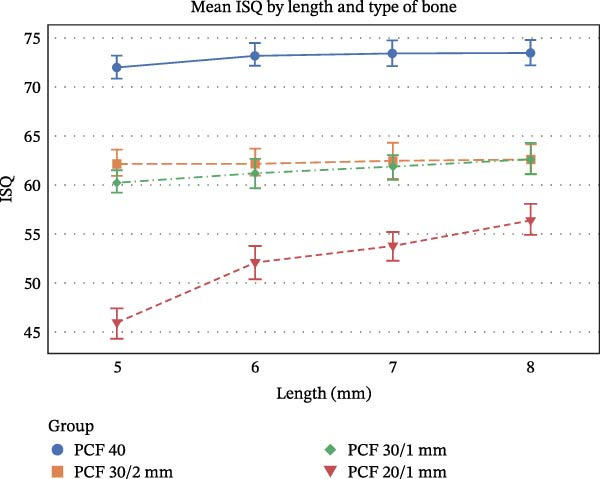
Mean ISQ values by bone density and implant length. The graph shows that stability (ISQ) is highly dependent on bone density. While the 40 PCF group maintains a high stability plateau across all lengths, the lower‐density 20 PCF group shows a distinct improvement in stability as implant length increases.

The group (bone type) effect was the dominant factor (*F* = 1159.41, *p*  < 0.001), followed by depth (*F* = 209.98, *p*  < 0.001). A significant interaction effect (*F* = 60.86, *p*  < 0.001) indicates that the rate of torque increase with depth was significantly steeper in the PCF 40 group than in the others (Figure 3). For ISQ, similar stability trends were observed, with significant main effects for group (*F* = 462.21, *p*  < 0.001) and depth (*F* = 15.23, *p*  < 0.001) (Figure 4). The interaction was also significant (*F* = 6.03, *p*  < 0.001), indicating that stability gains with depth varied by material density.

**Figure 5:**
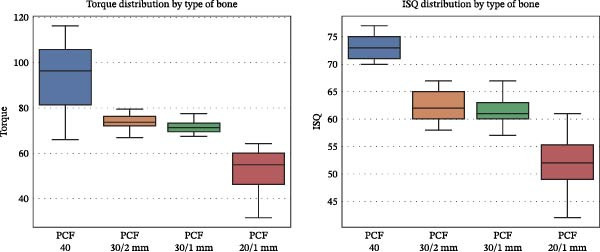
Box‐plot distribution of Torque and ISQ. Comparative box plots show the distribution of data across the four bone types. The 40 PCF‐CP group exhibits the highest median values for both metrics, whereas the 20 PCF‐CP1 group shows the lowest values and greatest variability in torque.

Tukey HSD testing confirmed that PCF 40 was statistically superior to all other groups for both torque and ISQ (*p*  < 0.05). No statistically significant difference was observed between PCF 30/1 mm and PCF 30/2 mm (*p* = 0.573 for Torque; *p* = 0.628 for ISQ), indicating that these two configurations yield comparable mechanical results (Figure 5).

**Figure 6:**
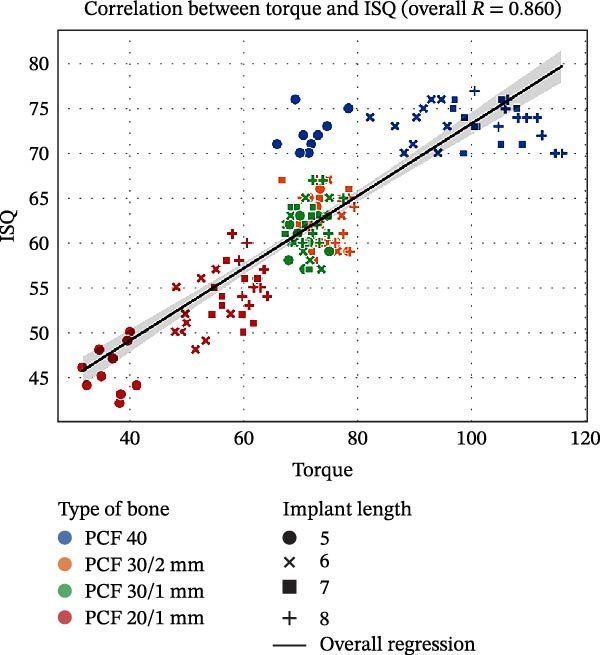
Correlation analysis between torque and ISQ. A scatter plot with a linear regression line demonstrates a strong positive correlation (*R* = 0.860, *p*  < 0.001) between the maximum insertion torque and the ISQ values. This suggests that mechanical resistance during placement is a reliable predictor of primary stability.

A strong overall positive correlation was found between Torque and ISQ (*r* = 0.860), indicating that higher insertion torque generally reflects higher primary stability. However, group‐specific analysis showed variation: PCF 20/1 mm maintained a high correlation (*r* = 0.788), whereas denser groups, such as PCF 40, showed a lower internal correlation (*r* = 0.199), likely because torque reached high values, while ISQ scores reached a biological/mechanical plateau (Figure 6).

## 4. Discussion

The assessment of dental implant stability is a cornerstone of modern implantology, heavily dictating the success of osseointegration and the predictability of immediate or early loading protocols. To address the clinical question of whether longer implants universally guarantee better primary stability, this study investigated how implant macrogeometry (specifically length) interacts with varying bone densities. By integrating RFA (ISQ) and ITV methodologies, this in vitro study provides a comprehensive evaluation of these biomechanical dynamics. The findings of the present study convincingly demonstrate that the biomechanical role of implant length is not universal; rather, its impact depends entirely on the structural quality of the receiving bone bed.

In low‐density medullary bone models with a thin cortical plate (PCF 20/1 mm), the data revealed a critical dependence on the implant length. In these scenarios, representing poor‐quality type D3 or D4 bone often found in the posterior maxilla, short implants (5 mm) yielded inadequate primary stability (ISQ = 45.8 and ITV = 36.8 Ncm). Such values historically suggest a higher risk of micro‐movement and potential implant failure under functional loading. However, simply increasing the implant length to 8 mm elevated the mechanical retention to clinically viable thresholds (ISQ = 56.3 and ITV = 61.36 Ncm). This confirms that in low‐density bone, where dense cortical anchorage is lacking, maximizing osteotomy depth allows greater apical and lateral engagement of the trabecular bone, thereby serving as the primary compensatory mechanism for poor bone quality [14].

Conversely, in highly dense, solid bone models (PCF 40, analogous to type‐D1 bone), primary stability was overwhelmingly high across all lengths. While increasing the implant length from 5 to 8 mm yielded a statistically significant increase in ITV (from 71.72–109.26 Ncm), the clinical relevance of this increase is debatable [6, 9]. An insertion torque exceeding 70 Ncm is already robust enough to support immediate loading protocols; pushing torque values beyond 100 Ncm in dense human bone could theoretically risk pressure necrosis and subsequent marginal bone loss. Therefore, it was hypothesized that in high‐density bone, shorter implants could be clinically advantageous, helping to avoid excessive surgical trauma while still ensuring excellent primary stability. Furthermore, in certain high‐density scenarios, the friction generated during the insertion of a longer implant may overprepare the soft trabecular space, effectively stripping the limited cortical anchorage by the time the implant is fully seated. This corroborates recent in vitro evaluations reporting distinct effects of bone density and length on primary stability across varying polyurethane block densities [12].

Perhaps the most intriguing finding was observed in the medium‐density substrates (PCF 30). Whether interacting with a 1 or 2 mm cortical layer, increasing the implant length from 5 to 8 mm produced a “plateau effect,” yielding no significant improvement in primary stability. This strongly suggests that in type D2 bone scenarios, stability is dictated almost entirely by the implant’s cervical engagement within the cortical plate [6, 9]. Once the cortical bone is fully engaged by the coronal threads, adding extra length to the softer underlying medullary bone provides a negligible mechanical benefit. This highlights the need for clinicians to select appropriate implant designs based on the patient’s specific bone characteristics rather than assuming that “longer is always better.” As demonstrated by some authors [14, 15], specific macrodesigns and deep threads often improve stability in challenging anatomical sites, suggesting that modifying the thread geometry, rather than increasing implant length, is the preferred strategy for D2/D3 bone types.

Clinically, these results challenge the outdated paradigm that “longer implants are always better.” Instead, they advocate for a site‐specific surgical approach. When clinicians encounter dense or medium‐density bone with adequate cortical thickness, using shorter implants can reduce surgical morbidity and anatomical risks (such as proximity to the sinus or nerves) without sacrificing primary stability. However, when preoperative imaging reveals poor‐density bone with minimal cortical support, utilizing a longer implant or modifying thread geometries to increase bone‐to‐implant contact becomes a biomechanical necessity to achieve the stability required for successful osseointegration [11].

## 5. Limitations of the Present Study

While this study provides clear biomechanical trends, its in vitro nature must be acknowledged. Polyurethane foam blocks (Sawbones) provide a highly standardized, reproducible medium that eliminates the anatomical and structural confounding variables of cadaveric or animal bone. However, they lack the biological vitality, vascularization, and anisotropic properties of the natural human bone. Specifically, while PCF blocks standardize density, they lack the complex, interconnected trabecular micro‐architecture and anisotropic behavior of in vivo human bone; therefore, direct extrapolation of these mechanical thresholds to clinical protocols should be interpreted with caution.

Furthermore, this study measures only primary (mechanical) stability and cannot evaluate the biological remodeling phase (secondary stability) or the host’s inflammatory response to high insertion torques. Finally, the uniform simulated cortical layers do not fully capture the complex and irregular cortical morphology encountered clinically. While previous in vitro studies have examined the device performance, only a few have assessed implants across different bone types. By utilizing a highly controlled, standardized substrate medium, this study successfully isolated the mechanical variables of length and density, eliminating operator technique and patient‐specific healing timelines as confounding factors [9, 11, 16]. Future in vivo studies and randomized clinical trials are needed to validate these biomechanical thresholds under dynamic functional loading.

## 6. Conclusions

The pursuit of optimal primary stability requires a nuanced understanding of the interactions between the implant macrogeometry and host bone architecture. Based on the findings of this in vitro evaluation and rejecting the null hypothesis formulated, the following conclusions can be drawn: (1) in low‐density bone with thin cortical plates, implant length is a critical determinant of success. Longer implants significantly increase primary stability, compensating for the lack of dense bone structure; (2) in medium‐density bone, primary stability is dictated predominantly by cortical engagement. Increasing implant length provides negligible mechanical benefit, suggesting that shorter implants are equally effective; and (3) in high‐density bone, while longer implants generate extreme insertion torques, even the shortest implants achieve excellent primary stability. Subject to future clinical validation, these findings hypothesize that clinicians might consider shorter implants in highly dense regions to potentially minimize the risk of pressure necrosis. Ultimately, implant length must be prescribed on a case‐by‐case basis, heavily guided by preoperative assessments of bone density to ensure predictable and successful clinical outcomes.

## Author Contributions

Sergio Alexandre Gehrke, Gracey Mosley, Juliana Campos Hasse Fernandes, and Gustavo Vicentis Oliveira Fernandes were responsible for conceptualization, methodology, software, validation, formal analysis, investigation, resources, data curation, writing – original draft, writing – review and editing, and visualization. Sergio Alexandre Gehrke and Gustavo Vicentis Oliveira Fernandes were responsible for supervision, and had full access to all data in this study and take full responsibility for the integrity and accuracy of the data analysis. Sergio Alexandre Gehrke was responsible for project administration.

## Funding

No funding was available for this study.

## Disclosure

All authors have read and approved the final version of the manuscript.

## Ethics Statement

The authors have nothing to report.

## Consent

The authors have nothing to report.

## Conflicts of Interest

The authors declare no conflicts of interest.

## Supporting Information

Additional supporting information can be found online in the Supporting Information section.

## Supporting information


**Supporting Information** Figure S1. Macrogeometry of the tested dental implants. The study utilized four tapered titanium implants of varying lengths: 5, 6, 7, and 8 mm. All implants feature an identical 5.0 mm diameter and a conical internal connection. Figure S2. Standardized synthetic bone blocks. Four variations of polyurethane foam blocks were used to simulate different bone densities and cortical thicknesses: (1) PCF 40: high‐density (D1) trabecular bone; (2) PCF 30/2 mm: medium‐density bone (D2) with 2 mm cortical thickness; (3) PCF 30/1 mm: medium‐density bone with 1 mm cortical thickness; and (4) PCF 20/1 mm: low‐density bone (D3/D4) with 1 mm cortical thickness.

## Data Availability

The authors confirm that the data supporting the findings of this study are available within the article and/or its Supporting Information section.
