# Supplementary figures and images for: Rethinking Implant Length: A Density‐Dependent Analysis of Primary Stability—An In Vitro Evaluation Using Resonance Frequency Analysis and Insertion Torque
Source: Int J Dent. 2026 Jun 19;2026:4201028. doi: 10.1155/ijod/4201028 (PMC13280807; doi:10.1155/ijod/4201028)

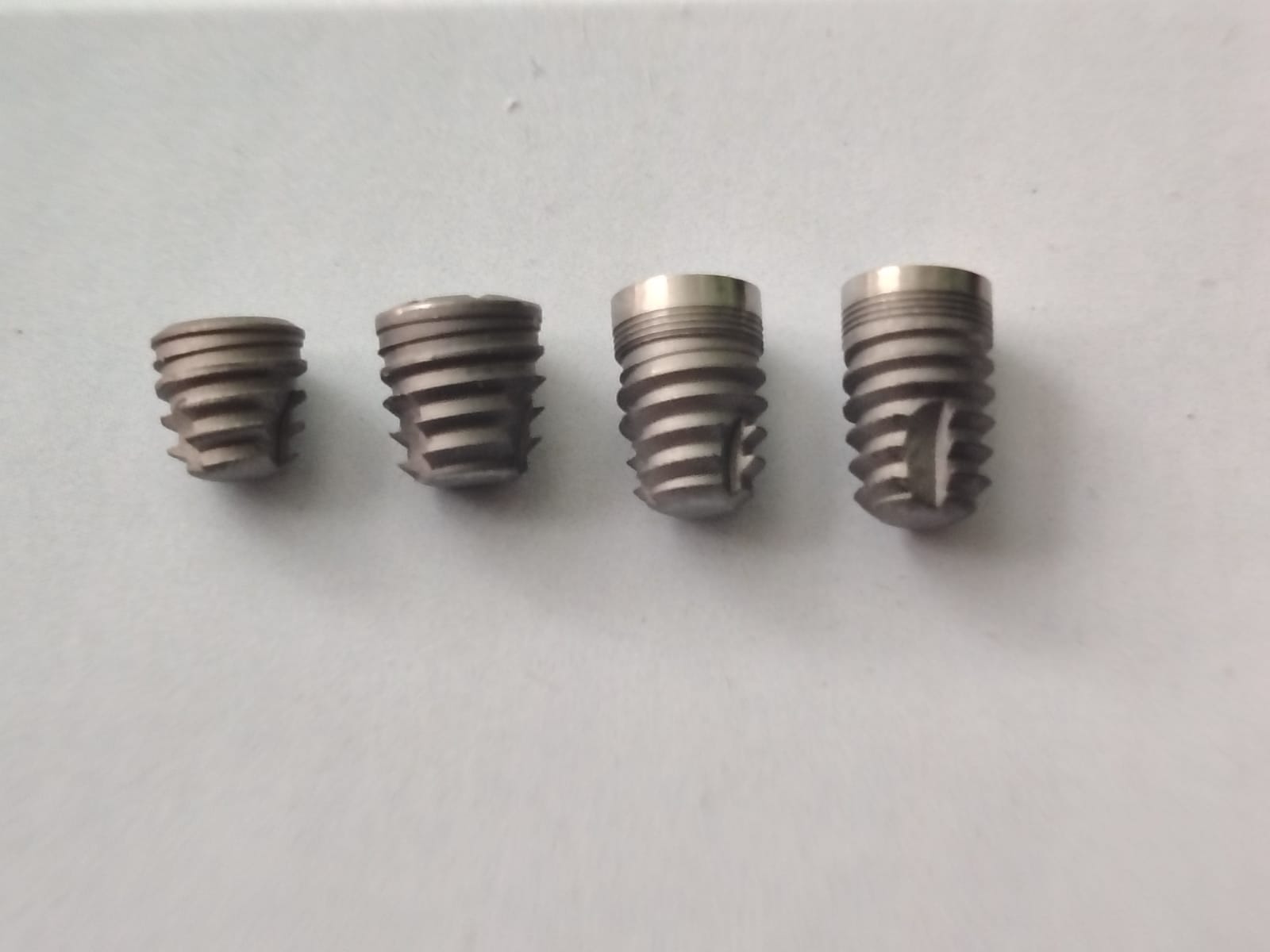

Supplement: Supplementary file 1 — Supporting Information Figure S1. Macrogeometry of the tested dental implants. The study utilized four tapered titanium implants of varying lengths: 5, 6, 7, and 8 mm. All implants feature an identical 5.0 mm diameter and a conical internal connection. Figure S2. Standardized synthetic bone blocks. Four variations of polyurethane foam blocks were used to simulate different bone densities and cortical thicknesses: (1) PCF 40: high‐density (D1) trabecular bone; (2) PCF 30/2 mm: medium‐density bone (D2) with 2 mm cortical thickness; (3) PCF 30/1 mm: medium‐density bone with 1 mm cortical thickness; and (4) PCF 20/1 mm: low‐density bone (D3/D4) with 1 mm cortical thickness. [file IJOD-2026-4201028-s001.zip › Suppl. Figure 1.jpg]

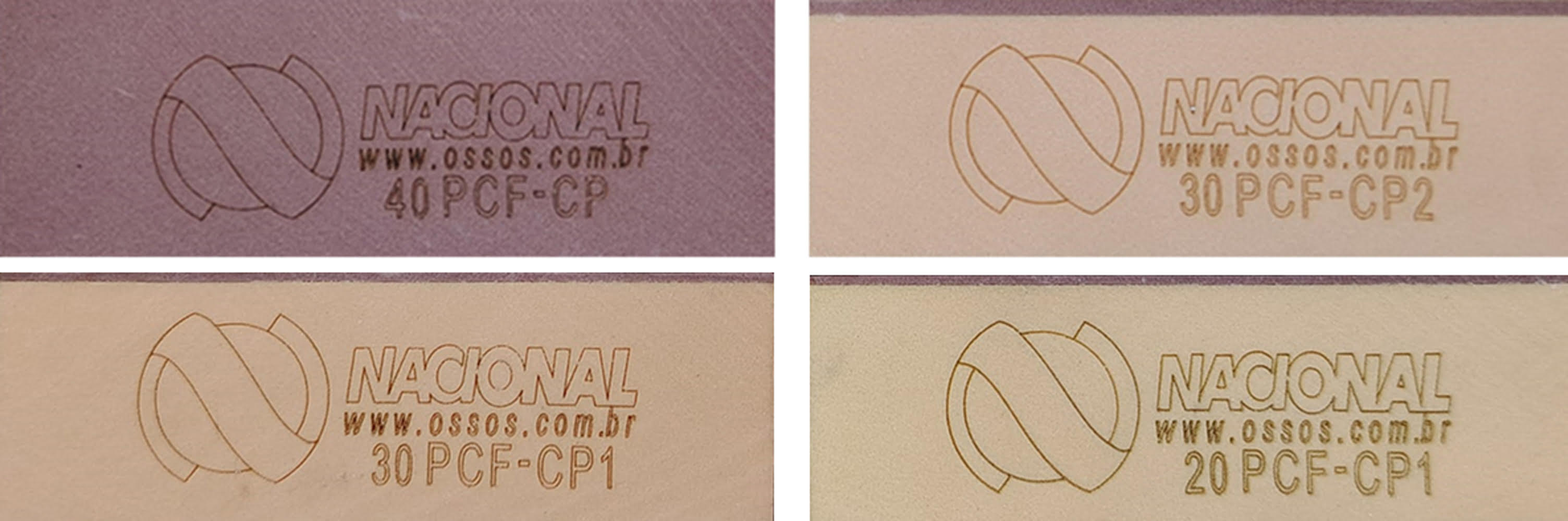

Supplement: Supplementary file 1 — Supporting Information Figure S1. Macrogeometry of the tested dental implants. The study utilized four tapered titanium implants of varying lengths: 5, 6, 7, and 8 mm. All implants feature an identical 5.0 mm diameter and a conical internal connection. Figure S2. Standardized synthetic bone blocks. Four variations of polyurethane foam blocks were used to simulate different bone densities and cortical thicknesses: (1) PCF 40: high‐density (D1) trabecular bone; (2) PCF 30/2 mm: medium‐density bone (D2) with 2 mm cortical thickness; (3) PCF 30/1 mm: medium‐density bone with 1 mm cortical thickness; and (4) PCF 20/1 mm: low‐density bone (D3/D4) with 1 mm cortical thickness. [file IJOD-2026-4201028-s001.zip › Suppl. Figure 2.jpg]
